# O-GlcNAc regulation of autophagy and α-synuclein homeostasis; implications for Parkinson’s disease

**DOI:** 10.1186/s13041-017-0311-1

**Published:** 2017-07-19

**Authors:** Willayat Y. Wani, Xiaosen Ouyang, Gloria A. Benavides, Matthew Redmann, Stacey S. Cofield, John J. Shacka, John C. Chatham, Victor Darley-Usmar, Jianhua Zhang

**Affiliations:** 1 Department of Pathology, Center for Free Radical Biology, University of Alabama at Birmingham, Birmingham, AL 35294-0017 USA; 2 Department of Biostatistics, University of Alabama at Birmingham, Birmingham, AL 35294-0022 USA; 3 Department of Pharmacology & Toxicology, University of Alabama at Birmingham, Birmingham, AL 35294-0019 USA; 4 Birmingham VA Medical Center, University of Alabama at Birmingham, Birmingham, AL 35294-0017 USA

**Keywords:** Autophagy, Parkinson’s disease, Proteasome, Rapamycin, α-synuclein, Mitochondria, mTOR, Akt, O-GlcNAcylation, Thiamet G

## Abstract

Post-translational modification on protein Ser/Thr residues by O-linked attachment of ß-N-acetyl-glucosamine (O-GlcNAcylation) is a key mechanism integrating redox signaling, metabolism and stress responses. One of the most common neurodegenerative diseases that exhibit aberrant redox signaling, metabolism and stress response is Parkinson’s disease, suggesting a potential role for O-GlcNAcylation in its pathology. To determine whether abnormal O-GlcNAcylation occurs in Parkinson’s disease, we analyzed lysates from the postmortem temporal cortex of Parkinson’s disease patients and compared them to age matched controls and found increased protein O-GlcNAcylation levels. To determine whether increased O-GlcNAcylation affects neuronal function and survival, we exposed rat primary cortical neurons to thiamet G, a highly selective inhibitor of the enzyme which removes the O-GlcNAc modification from target proteins, O-GlcNAcase (OGA). We found that inhibition of OGA by thiamet G at nanomolar concentrations significantly increased protein O-GlcNAcylation, activated MTOR, decreased autophagic flux, and increased α-synuclein accumulation, while sparing proteasomal activities. Inhibition of MTOR by rapamycin decreased basal levels of protein O-GlcNAcylation, decreased AKT activation and partially reversed the effect of thiamet G on α-synuclein monomer accumulation. Taken together we have provided evidence that excessive O-GlcNAcylation is detrimental to neurons by inhibition of autophagy and by increasing α-synuclein accumulation.

**Electronic supplementary material:**

The online version of this article (doi:10.1186/s13041-017-0311-1) contains supplementary material, which is available to authorized users.

## Introduction

Autophagy is important for recycling or removing damaged proteins and organelles in response to nutrient deprivation and stress [1, 2]. Deficient autophagy can lead to accumulation of dysfunctional intracellular proteins [3, 4] and have a significant impact on the pathogenesis and progression of neurodegenerative diseases [5]. The coupling of autophagy with metabolism is critical in neurons and may be related to their susceptibility to the proteotoxicity associated with neurodegenerative diseases such as Parkinson’s disease. A key nutrient sensing pathway is protein O-GlcNAcylation which is an evolutionarily conserved process in which proteins are reversibly modified at Ser/Thr residues in response to nutrient availability and stress [6–10]. The attachment of O-GlcNAc to proteins is catalyzed by O-GlcNAc transferase (OGT), and its removal by O-GlcNAcase (OGA) [11–13]. This protein post-translational modification process modifies Ser/Thr residues and can occur as rapidly as phosphorylation, thereby modulating protein function and signaling [10, 14–16]. Approximately 2–5% of glucose taken up by cells is consumed by the hexosamine biosynthetic pathway to generate uridine diphosphate N-acetyl-glucosamine (UDP-GlcNAc), the substrate for OGT [10, 11, 14–17].

The impact of O-GlcNAcylation has been studied in the context of neurodegenerative diseases, focusing mainly on O-GlcNAc modification of aggregation-prone proteins [18]. In the context of Alzheimer’s disease, O-GlcNAcylation may be beneficial because pharmacological inhibition of OGA increases tau O-GlcNAcylation, decreases tau phosphorylation, and decreases neurodegenerative phenotypes [19–24]. However, in a *C. elegans* model of neurodegeneration, an OGA inactive mutant that results in increased O-GlcNAcylation was shown to increase proteotoxicity [25]. In cell and fly models, increased O-GlcNAcylation has been shown to be associated with increased mutant huntingtin toxicity [26]. These observations suggest that changes in protein O-GlcNAcylation are an important contributor to the pathogenesis of neurodegenerative diseases but its effects are highly context-dependent [27]. Pertinent to Parkinson’s disease, it has been shown that α-synuclein, a protein involved in the pathophysiology of the disease, can be O-GlcNAcylated [28, 29]. It has been shown in vitro that O-GlcNAcylation at T72 decreases both the propensity of α-synuclein to aggregate and its toxicity when added to cultured cells [29]. Despite the fact that both autophagy and the O-GlcNAc pathway share nutrient and stress sensing properties, whether the O-GlcNAc pathway also contributes to autophagy regulation is only now being investigated [30, 31]. For example, it has shown in *C. elegans* and HeLa cells that O-GlcNAc modification of the protein SNAP-29 regulates autophagosome maturation [32]. We and others have previously shown that the O-GlcNAc pathway is active in the brain and that O-GlcNAcylated proteins are abundant in nerve terminals [12, 33–35]. O-GlcNAcylation levels in the brain have been shown to increase by 30% from 5 to 24 months, suggesting an involvement in age-dependent neuronal function [22, 33]. Furthermore, we have demonstrated that increased O-GlcNAc levels lead to impaired autophagic signaling and that key regulators of autophagy, Beclin-1 and Bcl-2, are O-GlcNAcylated in response to nutrient deprivation in cardiomyocytes [36]. In the present study we provide evidence that O-GlcNAcylation levels are significantly increased in Parkinson’s disease postmortem brains, and that pharmacological inhibition of OGA and thereby increasing O-GlcNAc levels in neuronal cultures decreases autophagic flux and induces α-synuclein accumulation.

## Results

Pharmacological inhibition of OGA by thiamet G increases O-GlcNAcylated proteins in primary neurons.

**Fig. 1:**
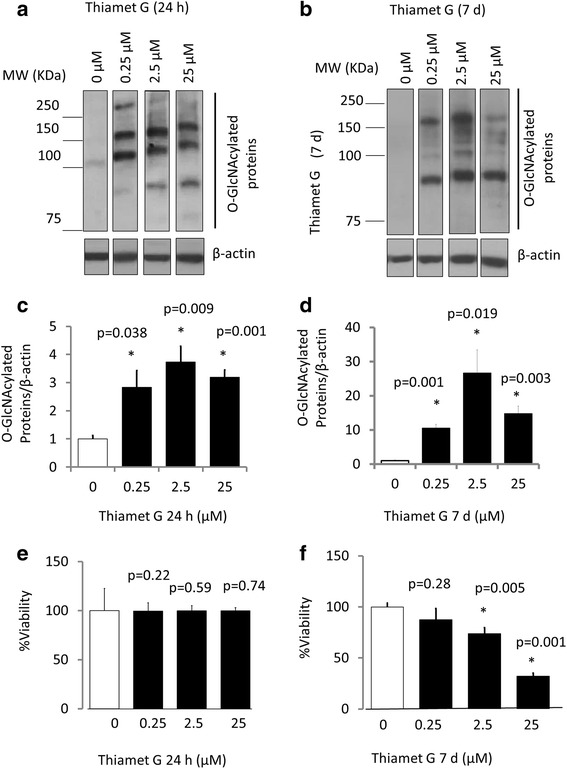
Inhibition of OGA by thiamet G (TG) increased O-GlcNAcylated protein levels in neurons. Primary cortical neurons were cultured from E18 rat embryos. At DIV7, neurons were exposed to TG (0.25, 2.5 and 25 μM) for 24 h (**a**) and 7 d (**b**). For detection of O-GlcNAc modified proteins, 10 μg protein was separated on a 7% SDS-PAGE and subjected to immunoblotting using CTD110.6 monoclonal antibody against O-GlcNAcylated proteins. β-actin was used as a loading control. (**c**-**d**) Quantification of the band intensities for O-GlcNAc modified proteins from (**a**-**b**) assessed with image J software 1.48e NIH, USA. (**e**-**f**) Cell viability was measured by the trypan blue exclusion method. For all panels, Data = mean ± SEM (*n* = 3), normalized to 0 μM TG. *p < 0.05 compared to 0 μM TG control, by ANOVA followed by Bonferroni’s Multiple Comparison post-hoc test. The exact *p* value is given above each bar for this comparison. The levels of protein O-GlcNAcylation among the 3 thiamet G concentrations were not significantly different

To determine whether increased protein O-GlcNAcylation alters neuronal survival we used thiamet G, a potent and highly selective inhibitor of O-GlcNAcase (OGA) [20]. Thiamet G is a competitive inhibitor of O-GlcNAcase with a Ki of 21 ± 3 nM. The functionally closest enzyme is lysosomal β-hexosaminidase, which has a Ki value for thiamet G of 750 ± 60 μM. Thus thiamet-G has 37,000-fold selectivity for OGA over the lysosomal β-hexosaminidase [20]. The primary rat cortical neurons were exposed to thiamet G over an acute (24 h) or chronic (7 d) time frame using a range of concentrations (0.25, 2.5 and 25 μM). Western blot analysis of the lysates demonstrated a significant increase in protein O-GlcNAcylation to a similar level at all three concentrations after 24 h exposure (Fig. 1a,c). At 7 d the overall level of O-GlcNAcylation relative to controls increased to approximately 25 fold at the 2.5 μM concentration, (Fig. 1a,d). No cell death was observed after a 24 h exposure to thiamet G at any concentration tested. However, after 7 d of exposure, significant cell death was observed at 2.5 and 25 μM thiamet G (Fig. 1e-f). Since subsequent experiments were designed to determine the cellular responses which occur in the absence of overt toxicity, the 0.25 μM concentration of thiamet G at 24 h or 7 d was used to assess both the acute and chronic effects on autophagy.

Prior studies in rat cardiac myocytes have demonstrated effects of high glucose on O-GlcNAcylation of mitochondrial proteins and impairment of complex I, III and IV activity in isolated mitochondria, which can be reversed by overexpression of OGA [37–40], how O-GlcNAcylation affects mitochondrial function in neurons is unknown. We observed that complex IV activity in the presence of ascorbate and TMPD was modestly decreased while complex I and II substrate linked oxidation in the presence of either ADP or FCCP was unchanged 24 h after thiamet G at 0.25 μM (Additional file 1: Figure S1A). Only after 7 d of thiamet G exposure, complex II substrate linked oxidation in the presence of FCCP was also decreased (Additional file 1: Figure S1B). A mitochondrial stress test on intact cells after either 24 h or 7 d of thiamet G exposure demonstrated that in intact cells, no significant changes occurred in basal, maximal, ATP-linked, proton leak and non-mitochondrial oxygen consumption rates (data not shown). Furthermore, thiamet G did not affect mitochondrial DNA copy number (Additional file 1: Figure S1C); or overall mtDNA damage (Additional file 1: Figure S1D). There was also no change in PGC1α after either 24 h or 7 d thiamet G exposure (Additional file 1: Figure S1E–F), suggesting mitochondrial biogenesis is not affected. Therefore, the levels of protein O-GlcNAcylation in response to the sub-lethal thiamet G concentration over the time period in our experiments are not sufficient to decrease mitochondrial quality.

### OGA inhibition decreases autophagic flux in primary neurons

**Fig. 2:**
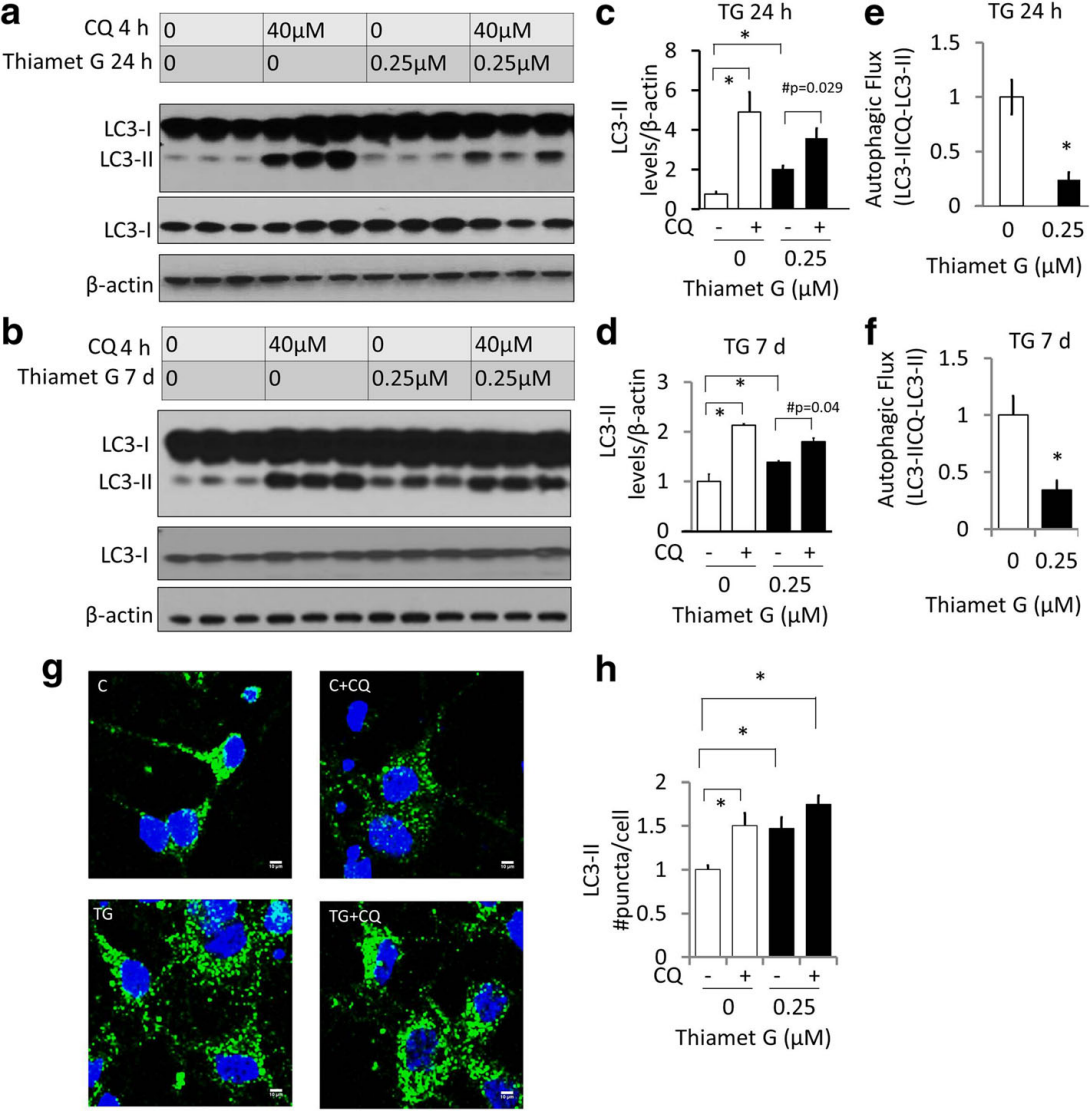
Inhibition of OGA by thiamet G (TG) decreases autophagic flux. (**a**&**b**) Western blot analysis of protein extracts obtained from rat cortical neurons following exposure to thiamet G (0.25 μM) for 24 h (**a**) and 7 d (**b**) in the presence or absence of chloroquine (CQ). For detection of LC3, 5 μg protein was separated on a 12% SDS-PAGE and subjected to immunoblotting with anti-LC3 antibody. β-actin was used as a loading control. (**c-d)** Bar graphs represent the quantification of the band intensities for LC3II using image J software 1.48e NIH, USA. (**e-f**) The calculated values of LC3-II with CQ minus LC3-II without CQ (Autophagic Flux) from **a-b** and normalized to 0 μM TG. (**g-h**) LC3 puncta were detected by immunocytochemistry and quantified as puncta/cell. Scale bar = 10 μm. For all panels, Data = mean ± SEM (*n* = 3). *p < 0.05 compared to 0 μM thiamet G without CQ. For Panel C-D, #*p* < 0.05 compared to without CQ. Results were analyzed by ANOVA followed by Bonferroni’s Multiple Comparison post-hoc test

To determine whether autophagy is altered by OGA inhibition, we exposed cortical neurons to thiamet G (0.25 μM) for 24 h or 7 d and measured levels of LC3-II. LC3-II is the lipidated form of LC3-I and is associated with autophagosomes and therefore can be used as a marker of autophagosomal mass [41]. We found that exposure to 0.25 μM thiamet G resulted in an increase in LC3-II levels (approx. 2 x fold after 24 h and 1.3 x fold after 7d) (Fig. 2a-d). To determine whether this was due to inhibition of autophagic flux co-incubation with the lysosomal inhibitor chloroquine (CQ) was used, which increases LC3-II level by blocking autophagy completion. The differences in LC3-II levels in the presence and absence of CQ are proportional to autophagosome recycling by lysosomes, and is termed autophagic flux [41]. Increased autophagic flux induced by a pharmacological treatment is defined by an increase in LC3-II in combination with chloroquine. Conversely a decrease in LC3-II in combination with CQ can be ascribed to a decrease in autophagic flux [2, 41]. As expected the addition of CQ increased the control levels of LC3-II (5 x fold at 24 h and 2 x fold at 7d), representing the maximal autophagic flux. In the presence of thiamet G, CQ increased LC3-II levels to a lesser extent (Fig. 2a-d). As a quantitative measure of autophagic flux, the difference in LC3-II levels in the presence and absence of CQ were then calculated (LC3IICQ-LC3II) (Fig. 2e-f). As shown, autophagic flux in response to thiamet G is approximately 24% of control levels at 24 h and 34% at 7 d. As an additional measure of autophagic flux, immunocytochemistry was used to assess LC3 puncta in the presence of thiamet G (24 h) with and without CQ (4 h). Consistent with the western blot data, thiamet G increases LC3 puncta in the absence of CQ. CQ also increased LC3 puncta in control but did not increase further in the presence of thiamet G (Fig. 2g-h). Prior studies have found that the autophagy and proteasomal pathways may interact [42, 43] and that O-GlcNAcylation has been shown to decrease proteasome activities [44]. In this study with primary neurons, we found that after either 24 h or 7 d of thiamet G, there was no decrease of proteasomal activities. There was also no change of overall ubiquitinated proteins under these conditions (Additional file 2: Figure S2).

### Increased protein O-GlcNAcylation in Parkinson’s disease postmortem brains

**Fig. 3:**
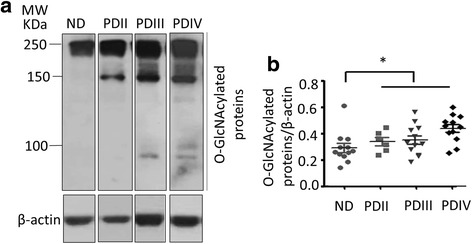
Protein O-GlcNAcylation in Parkinson’s disease postmortem brains. (**a**) Western blot analysis of protein extracts obtained from postmortem temporal cortical samples obtained from non-disease (ND) and stage II-IV Parkinson’s disease (PDII, PDIII and PDIV) specimens. 10 μg protein was separated on a 7% SDS-PAGE, and subjected to immunoblotting using CTD110.6 monoclonal antibody against O-GlcNAcylated proteins. β-actin antibody was used as a loading control. (**b**) Quantification of the band intensities for O-GlcNAc modified proteins from (**a**) using image J software 1.48e NIH, USA. *n* = 6–12 samples. There is a significant difference in median (*p* = 0.0095), with PDIV, PDIII, and PDII compared to ND (*p* = 0.0086, 0.0120, 0.0351, respectively, Wilcoxon). The *p* value corrected for multiple tests are: *p* = 0.0258, 0.0360, and 0.1053, respectively. There were no significant differences between different stages of PD by ANOVA (PDII-IV stage). * *p* = 0.0095

To determine whether O-GlcNAcylation levels are altered in Parkinson’s disease, we analyzed postmortem temporal cortex specimens from both Parkinson’s disease brains (Parkinson’s disease stage PDII, III and IV by Lewy body pathology (45)) and no-disease (ND) age-matched controls by western blot analysis using a monoclonal antibody recognizing O-GlcNAcylated proteins (Fig. 3a, Additional file 3: Figure S3). We found that there was a significant increase in median O-GlcNAc levels with PDIV, PDIII, and PDII staged samples compared to ND (Fig. 3b).

### Inhibition of OGA increases α-synuclein levels in primary neurons

**Fig. 4:**
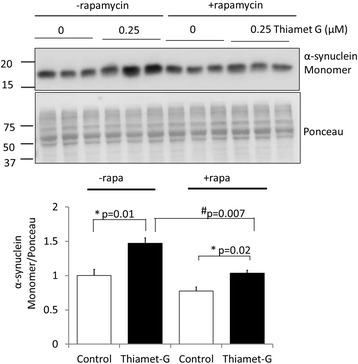
OGA inhibition by thiamet G (TG) increases and α-synuclein accumulation, this can be partially reversed by MTOR inhibition by rapamycin. DIV7 primary rat cortical neurons were exposed to rapamycin (1 μM) and/or TG (0.25 μM) for 7 d. Western blot analyses of α-synuclein were performed. Ponceau staining was used as a loading control. Inhibition of OGA increased, and inhibition of MTOR decreased, α-synuclein levels. α-synuclein monomer is increased by TG, and decreased in TG + rapamycin compared to TG. Quantification of western blot band intensities was performed using NIH image J. *p < 0.05 compared to no TG, #*p* < 0.05 compared to –rapamycin, *n* = 3. Results were analyzed by ANOVA followed by Bonferroni’s Multiple Comparison post-hoc test

To determine whether inhibition of O-GlcNAc removal affects α-synuclein accumulation, a phenomenon associated with Parkinson’s disease pathologies, we performed western blot analyses of α-synuclein in primary neurons exposed to thiamet G. We found that thiamet G exposure increased monomeric α-synuclein compared to control after 7 d of thiamet G exposure by ~50% (Fig. 4), but not after 24 h (Data not shown). To determine whether an activation of autophagy can attenuate the thiamet G-induced increase in α-synuclein monomer, we exposed cells to the MTOR inhibitor rapamycin. We found that rapamycin did not change the basal levels of α-synuclein in the absence of thiamet G, but attenuated the thiamet G-dependent effect on α-synuclein accumulation by 48% (Fig. 4).

### Inhibition of OGA increases MTOR activation

To investigate the possible mechanisms of thiamet G-dependent inhibition of autophagy, we first performed real-time RT-PCR analyses of selective autophagy and lysosomal genes. We found that there was a modest increase in *p62* mRNA at 24 h but not at 7 d, although p62 protein levels remain unchanged (Additional file 4: Figure S4, Additional file 5: Figure S5). There was no change of *Becn* mRNA after 24 h or 7 d thiamet G exposure (Additional file 4: Figure S4A-B), while a modest increase of BECN protein was seen after 7 d (Additional file 4: Figure S4E-F, 4H). mRNA and protein levels of *Hsc70* and *Lamp2a*, which are involved in chaperone mediated autophagy, were unchanged (Additional file 4: Figure S4A-B, 4I–L), and there was no change in *Lamp1* mRNA (Additional file 4: Figure S4A-B).

**Fig. 5:**
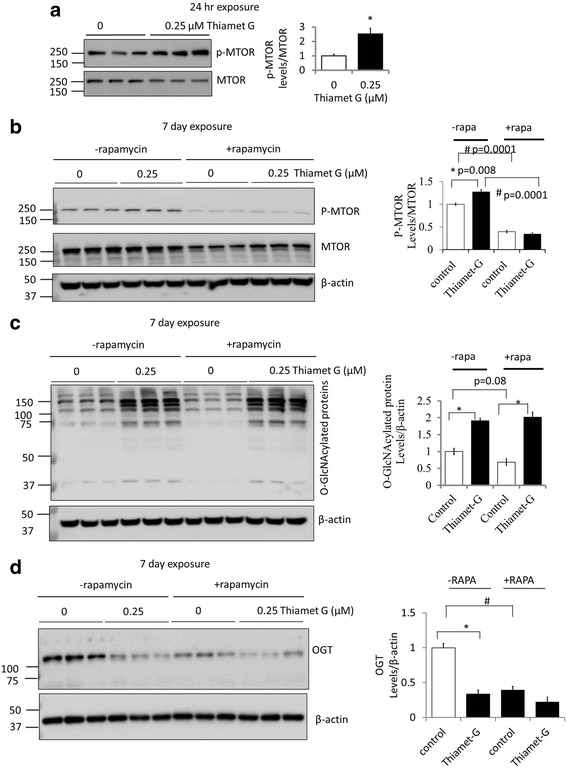
OGA inhibition by thiamet G (TG) increases MTOR activation. (**a**) Primary cortical neurons were cultured from E18 rat embryos. At DIV7, neurons were exposed to TG (0.25 μM) for 24 h. Western blot analyses were performed with anti-p-MTOR (at Ser2448) and anti-MTOR antibodies. Bar graphs represent respective quantifications. (**b-d**) DIV7 primary rat cortical neurons were exposed to rapamycin (1 μM) and/or TG (0.25 μM) for 7 d. Western blot analyses were performed. β-actin was used as a loading control. (**b**) Inhibition of OGA by TG (7 d) increased p-MTOR/total MTOR and rapamycin decreased it. Western blot analyses of p-MTOR (at Ser2448), and MTOR were performed. (**c**) TG (7 d) increased total O-GlcNAcylated proteins even in the presence of rapamycin. Western blot analyses of O-GlcNAcylated proteins were performed. (**d**) Thiamet G and rapamycin both decreases O-GlcNAc transferase (OGT), as shown by the western blot analyses. Quantification of western blot band intensities was performed using NIH image J. For all panels, Data = mean ± SEM (*n* = 3), *p < 0.05 compared to no TG, #*p* < 0.05 compared to –rapamycin. Results were analyzed by ANOVA followed by Bonferroni’s Multiple Comparison post-hoc test

In contrast, we observed that thiamet G increased p-MTOR (Ser2448)/total MTOR 2.5 fold after 24 h (Fig. 5a) and also at 7 days albeit to a lesser extent (Fig. 5b). We then tested the impact of rapamycin in the presence and absence of thiamet G. As shown in Fig. 5b rapamycin decreased MTOR phosphorylation not only in the control, but also in the presence 0.25 μM thiamet G. Interestingly, rapamycin alone modestly (32%) decreased overall O-GlcNAcylated protein levels (*p* = 0.08) but not in the presence of thiamet G (Fig. 5c). To investigate this aspect further we examined the levels of the enzyme which adds the O-GlcNAc moiety to proteins, O-GlcNAc transferase (OGT). As shown in Fig. 5d, the levels of OGT decrease in the presence of thiamet G, likely due to a compensatory response to inhibition of OGA which is consistent with previous report in several human cell lines [45]. Interestingly, we found that rapamycin also decreased OGT protein levels, as has been reported in HepG2 cells and ascribed to an effect of MTOR on OGT protein stability in the cell [46, 47].

### Inhibition of OGA decreases autophagic flux even in the presence of rapamycin

**Fig. 6:**
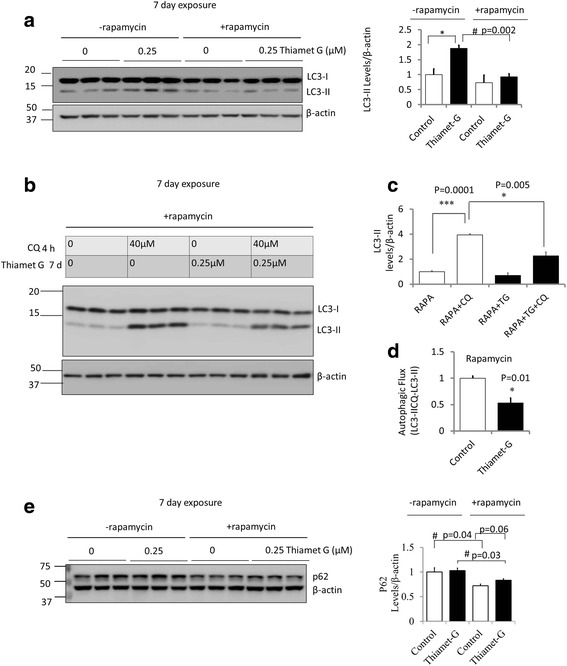
Inhibition of OGA by thiamet G (TG) decreased autophagic flux even in the presence of rapamycin. (**a**) DIV7 primary rat cortical neurons were exposed to rapamycin (1 μM) and/or TG (0.25 μM) for 7 d. Western blot analyses of LC3 were performed. The increase of LC3-II by thiamet G was attenuated by rapamycin. (**b**) Western blot analyses of LC3-II were performed in the presence and absence of chloroquine (CQ) as described in Fig. 2. (**c**) Quantification of western blot band intensities was performed using NIH image J. (**d**) Calculated values of LC3-II with CQ minus LC3-II without CQ (Autophagic Flux) from **b-c** and normalized to 0 μM TG control. (**e**) DIV7 primary rat cortical neurons were exposed to rapamycin (1 μM) and/or TG (0.25 μM) for 7 d. Western blot analysis of p62 was performed. Inhibition of mTOR decreased p62. For all panels, β-actin was used as a loading control, Data = mean ± SEM (*n* = 3), *p < 0.05 compared to no TG, #*p* < 0.05 compared to –rapamycin. Results were analyzed by ANOVA followed by Bonferroni’s Multiple Comparison post-hoc test. *** *p *= 0.0001

To determine whether thiamet G effects on autophagic flux are dependent on MTOR phosphorylation we measured autophagic flux in the presence of rapamycin after 7 d exposure to both agents. We found that as shown previously in Fig. 2d thiamet G induced accumulation of LC3-II which was attenuated in the presence of rapamycin (Fig. 6a). In the presence of rapamycin, CQ induced LC3-II accumulation by 3.9 x fold which is substantially greater than in the absence of rapamycin (Figs. 2d, 6b-c). The increase by CQ in the presence of both rapamycin and thiamet G is decreased from that in the presence of rapamycin alone from 3.9 x fold to 2 x fold (Fig. 6b-c) consistent with thiamet G decreasing autophagic flux (Fig. 6d). These data suggest that the overall rapamycin-dependent increase in autophagic flux persists on the background of thiamet G. Consistent with an effect of rapamycin in activating autophagy, we found that p62 is decreased by rapamycin both in the presence and in the absence of thiamet G, while there is a trend (*p* = 0.06) of less decrease of p62 by rapamycin in the presence of thiamet G, consistent with a decreased autophagic flux by thiamet G (Fig. 6e).

### MTOR activation and AKT signaling

**Fig. 7:**
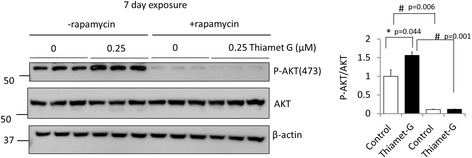
Thiamet G increases and rapamycin decreases AKT phosphorylation. DIV7 primary rat cortical neurons were exposed to rapamycin (1 μM) and/or TG (0.25 μM) for 7 d. Inhibition of OGA (7 d) increased p-AKT (S473) and inhibition of MTOR decreased p-AKT, as shown by the western blot analyses. β-actin was used as a loading control. Quantification of western blot band intensities was performed using NIH image J. Data = mean ± SEM (*n* = 3), *p < 0.05 compared to no TG, #*p* < 0.05 compared to –rapamycin. Results were analyzed by ANOVA followed by Bonferroni’s Multiple Comparison post-hoc test

Since it has been shown that AKT regulates MTOR activation [48, 49], and that AKT can be O-GlcNAcylated [50], we investigated whether this pathway is regulated by O-GlcNAc in primary neurons. Indeed, we have found that thiamet G increased p-AKT in primary neurons (Fig. 7). Interestingly, rapamycin decreased p-AKT in both control and thiamet G groups in primary neurons (Fig. 7), suggesting that MTOR activity is required for AKT phosphorylation or rapamycin, an observation more consistent with the report that AKT is inactivated in response to an inhibitor of both MTORC1 and MTORC2, and prolonged rapamycin treatment in certain cell types [49, 51, 52].

## Discussion

In this study we investigated the role of O-GlcNAcylation on neuronal autophagy and α-synuclein homeostasis. We show that inhibition of O-GlcNAc removal in primary neurons attenuates autophagic flux. Over time, hyper-O-GlcNAcylation also causes α-synuclein monomer accumulation prior to cell death, while mitochondrial function is largely intact. Of importance, we report for the first time an increase in global protein O-GlcNAcylation in postmortem temporal cortical samples obtained from Parkinson’s disease patients compared to controls. Since α-synuclein inclusions do not appear in the temporal cortex until stage IV, our results suggest that increased overall O-GlcNAcylation precedes α-synucleinopathy.

In this study we inhibited O-GlcNAc removal acutely using a highly specific inhibitor of O-GlcNAcase, thiamet G [20]. Although this is a highly specific inhibitor, off-target effects cannot be ruled out and future studies using a conditional knockout of OGA, or overexpressing OGT in neurons in vivo are planned. We found no changes of overall ubiquitinated proteins nor proteasome activities after 24 h or 7 d of thiamet G, suggesting that neurons rely more heavily on macroautophagy rather than the proteasome for clearance of damaged proteins. Our observation that both increased MTOR activation and increased LC3-II accumulation are associated with thiamet G exposure suggests that thiamet G inhibits both initiation and completion of autophagy. This observation is further substantiated by the decreased autophagic flux in rapamycin exposed neurons in the presence of thiamet G. Regarding mechanisms of autophagy regulation, although our study focused on effects of inhibition of OGA on MTOR activation, it is conceivable that O-GlcNAc modification of SNAP-29 may also occurs in neurons to regulate autophagosome maturation [32]. Future studies with sensitive and quantitative methods such as mass spectrometry are needed to determine whether SNAP-29 O-GlcNAcylation also occurs in neurons and contributes to α-synuclein accumulation in response to thiamet G.

At present the exact mechanisms of how thiamet G increases both MTOR and AKT phosphorylation and how rapamycin decreases both MTOR and AKT phosphorylation are not well defined. One possibility is that increasing O-GlcNAc levels activate MTOR through Sp1 activation of transcription of glycerol-3-phosphate acyltransferase-1 (GPAT1) which activates MTOR [53]. Whether in neurons thiamet G induced MTOR activation is mediated by Sp1 activation of GPAT1 will need to be further investigated. In addition to being activated by MTOR inhibition [54], AKT-S473 phosphorylation has been shown to inhibit autophagosome formation via phosphorylation of BECN [55].

O-GlcNAcylation has been shown to play a protective role against tau phosphorylation in HEK cells, rats and transgenic mice carrying pathogenic tau [21, 24]. While these prior findings support the potential benefit of developing pharmacological agents that increase O-GlcNAcylation and thereby decreasing tau phosphorylation for the treatment of Alzheimer’s disease, our observations that α-synuclein accumulates in response to thiamet G suggest that such an approach may be detrimental to α-synuclein homeostasis. This is important as α-synuclein accumulation occurs in ~60% of Alzheimer’s disease cases and in the majority of Parkinson’s disease cases. We found that in the presence of thiamet G, overall protein O-GlcNAcylation is not decreased by rapamycin, whereas α-synuclein and p62 are decreased, suggesting that rapamycin-dependent increase in autophagic flux is sufficient to overcome the inhibitory effects of increased O-GlcNAcylation on autophagy of specific substrates such as α-synuclein and p62. This finding has important implications for the clinical applications of rapamycin since rapamycin can be beneficial in decreasing α-synuclein even in the presence of pathologic suppression of autophagy with increased O-GlcNAcylation.

## Conclusion

**Fig. 8:**
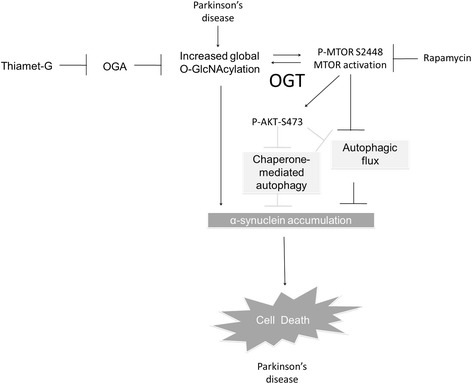
Working model. In our study we demonstrated that Parkinson’s disease postmortem brains exhibit increased O-GlcNAcylated proteins. Increasing O-GlcNAcylated proteins by inhibition of OGA, the enzyme responsible for O-GlcNAc removal, resulted in MTOR activation, AKT phosphorylation, and attenuation of autophagic flux. Prolonged inhibition of OGA increased α-synuclein accumulation and resulted in cell death. Prior studies suggest that an increase in AKT phosphorylation may attenuate autophagy by phosphorylating BECN and attenuate chaperone mediated autophagy by modulating translocation complex stability. Rapamycin inhibits MTOR activation and can partially reverse thiamet G effects on p62 and α-synuclein accumulation

In conclusion, the present study shows several key findings as summarized in Fig. 8. Thiamet G inhibits OGA, leading to increased O-GlcNAcylated proteins despite decreased OGT levels and suggests that removal of O-GlcNAc moiety from proteins is the critical step in controlling protein O-GlcNAcylation levels in neurons. The consequences of this, over a 7 day period of time, are activation of MTOR and AKT, attenuation of autophagic flux, and an increase in endogenous α-synuclein. Inhibition of MTOR decreases AKT phosphorylation, decreases p62 and α-synuclein accumulation induced by thiamet G, but did not decrease overall O-GlcNAcylation. This finding is pertinent to Parkinson’s disease, since α-synuclein accumulation has been shown in neurons and neurites in Parkinson’s disease postmortem brains and we found an elevation of O-GlcNAcylated proteins in Parkinson’s disease postmortem brains. Furthermore, since α-synuclein accumulation also occurs in Dementia with Lewy Bodies and Alzheimer’s disease, the observation may have even broader implications beyond Parkinson’s disease. These studies lay the foundation for further investigating the interaction between O-GlcNAcylation-MTOR-AKT-autophagy and α-synuclein homeostasis, for identification of O-GlcNAc targets and in turn help design competitive peptides to prevent O-GlcNAc modification-induced autophagic inhibition and α-synuclein accumulation.

## Method

### Chemicals

Neurobasal medium (21103–049), B-27 supplement (17504044–044), L-glutamine (25030–081) and penicillin-streptomycin (15140–122) were purchased from Life Technologies. Chloroquine (C6628), oligomycin (75351), FCCP (C2920), rotenone (R8875), antimycin-A (A8674), pyruvate (P5280), malate (M6413), ADP (A2754), succinate (S2378), L-ascorbic acid (A5960), N,N,N′,N′-Tetramethyl-p-phenylenediamine (TMPD; T7394), poly-L-lysine, rapamycin (R8781) were all purchased from Sigma. PMP (102504) was from Seahorse Bioscience. Thiamet G was purchased from SD Chem Molecules LLC. (Owings Mills, MD). Papain was from Worthington.

### Postmortem brain analyses

Temporal cortex specimens from No-disease (ND) controls and Parkinson’s disease (PDII-IV stage) were obtained from the Arizona Parkinson’s disease Consortium (Dr. Thomas G. Beach, Director) and the Michael J Fox Foundation, classified by a unified staging system [56, 57]. Robust cortical synucleinopathy is evident at stage IV. Tissues were lysed in lysis buffer containing 50 mM Tris pH 7.4, 175 mM NaCl, 5 mM EDTA pH 8.0. The lysates were centrifuged at 10,000 x g for 15 min at 4 °C. Supernatants were subjected to BCA protein assay, and 15 μg protein was loaded per lane for western blotting analyses.

### Primary neuron cultures

Primary rat cortical neurons were obtained from embryonic day 18 embryos [58, 59]. Timed-pregnant Sprague–Dawley rats (Charles River Laboratories, Wilmington, MA, USA) were sacrificed by CO_2_ inhalation and embryos were collected in a petri dish and placed on ice. Dissections were performed in ice-cold Hanks’ balanced sodium salts (without Ca^2+^ and Mg^2+^). Cerebral cortices were isolated and collected in 15-ml Falcon tubes. The tissues were incubated for 15 min at 37 °C with papain (Worthington), followed by mechanical dissociation by pipetting. Cells were briefly exposed to DNase I and finally concentrated by centrifugation at ~200 x g for 5 min and resuspended in Neurobasal medium containing 2% B27 supplement (Invitrogen), 1% Pen–Strep (10,000 U/ml, 10,000 mg/ml), and 0.5 mM L-glutamine. Cell preparations were plated in XF96 plates and 48-well plates coated with 0.1 mg/ml poly-L-lysine (Sigma) for mitochondrial bioenergetics studies and western blotting respectively. The cultures were kept in a humid incubator (5% CO_2_, 37 °C). 7 d in vitro (DIV7) cultures were used for all experiments. All animal studies have been approved by IACUC University of Alabama at Birmingham.

### Western blot analysis

250,000 cells were seeded on poly-L-lysine coated 48-well plates and treated for either 24 h or 7 d with different concentrations of thiamet G (0.25–25 μM). Then cells were lysed in RIPA buffer (150 mM NaCl, 1% Triton X-100, 2 mM EDTA, 0.1% SDS, 50 mM Tris, pH 8.0), and protein extracts were separated by SDS-PAGE and probed with the antibodies listed below. Since we had a lot of samples from human postmortem brains, we had to run more than one gels. Thus, all the samples for the control and disease conditions were run at the same time on two separate gels using a common loading control to compare between the 2 gels for quantification. Relative levels of protein were quantified using Image J software from the NIH (Bethesda, MA, USA) and normalized to loading control.

### Primary antibodies

O-GlcNAcylated proteins (UAB hybridoma core, CTD110.6,1:1000), p62 (Abnova, H00008878-M02,1:2000), Microtubule-associated protein 1 light chain 3 alpha/LC3 (Sigma, L8918, 1:2000), β-actin (Sigma, A5441, 1:5000), OGT (Sigma, O6264, 1:2000), α-synuclein (Santa Cruz, sc7011R, 1:1000), PGC1α (Santa Cruz, sc13067,1:1000), HSC70 (abcam,19,136–100, 1:5000), LAMP2a (abcam, 37,024, 1:1000), Ubiquitin (DAKO, Z0458, 1:1000), GAPDH (Millipore, MAB374,1:5000), MTOR (Cell Signaling, 2983S, 1:1000), p-MTOR-S2448 (Cell Signaling 2971S, 1:1000), p-AKT-S473 (cell signaling, 587F11, 1:1000), AKT (cell signaling, 4691S,1:1000).

### Secondary antibodies

Goat anti-rabbit IgG-HRP (sc2004, 1:5000), Goat anti-mouse IgG-HRP (sc2005, 1:5000), Goat anti-mouse IgM-HRP (Calbiochem, 1:10,000) and Goat anti-rat IgG-HRP (sc-2006, 1:5000).

### Mitochondrial respiration in intact and permeabilized neurons

Respiration in intact neurons was measured using a Seahorse XF96 Analyzer (Seahorse Bioscience) [60–62]. Cells were switched to XF media 30 min before measurement of oxygen consumption rate (OCR), followed by sequential injection of oligomycin (1 μg/ml), FCCP (1 μM), and antimycin A (10 μM) and measurements of OCR between injections. For the mitochondrial activity assay cells were switched to MAS buffer (70 mM Sucrose, 220 mM mannitol, 10 mM KH_2_PO4, 5 mM MgCl_2_, 2 mM HEPES, 1 mM EGTA, adjusted pH to 7.2) [63], OCR was measured after injection with 20 μg/ml PMP (1 nM) to permeabilize the cells. Initially, complex I substrates were included with the PMP plus FCCP for complex I activity assays, then injected with 2 μM rotenone to inhibit complex I activity, followed by injection of complex II substrates then injected with Antimycin A. In a separate assay, complex IV activity was measured in PMP permeabilized neurons by injection of ascorbate and TMPD, followed by sequential injections of rotenone and azide. Complex I activity was calculated as the difference in OCR before and after rotenone injection. Complex II activity was calculated as the difference between before and after antimycin injection. Complex IV activity was calculated as the difference after ascorbate injection and after azide injection, *n* = 3–6 independent incubations. The experiments were repeated with >3 independent cultures and data are presented as representative experiments.

### Assessment of cell viability

Primary cortical neurons were plated at 80,000 cells per well in 96-well plates, and following treatment, trypsinized and resuspended in Neurobasal media. Trypan blue was added, and the cells non-permeable to trypan blue were counted as viable [64, 65].

### Immunocytochemistry

Autoclaved glass coverslips were placed in 24-well culture plates and seeded with primary cortical rat neurons at a density of 240,000 cells per well. Cells were exposed to thiamet G and/or chloroquine for 24 h, followed by fixation with 4% paraformaldehyde and 4% sucrose. After fixation, cells were blocked with 5% BSA and 10% horse serum and then probed for LC3 (Sigma L8918). AlexaFluor 488 and Hoechst were added before slides were mounted and visualized with a Leica TCS SP5 V confocal laser scanning microscope. Cells with LC3 puncta were then counted.

### Mitochondrial copy number

The mitochondrial and nuclear 18S DNA were amplified using RT Real-Time SYBR Green PCR master mix (Invitrogen) in an ABI 7500 PCR machine [66]. The primer sequences used for mtDNA were mtDNA-F (5′-CCAAGGAATTCCCCTACACA-3′) and mtDNA-R (5′- GAAATTGCGAGAATGGTGGT-3′). The primer sequences for the nuclear DNA were 18S–F (5′-CGAAAGCATTTGCCAAGAAT-3′) and 18S–R (5′-AGTCGGCATCGTTTATGGTC-3′) and targeted nuclear 18S DNA. Cycling conditions were as follows: 94 °C for 15 s, followed by 40 cycles at 94 °C for 15 s, 60 °C for 1 min, then and 72 °C for 10 min. The mtDNA copy number was normalized to the amplification of the 18S nuclear amplicon.

### Mitochondrial DNA damage

Mitochondrial DNA damage (mtDNA) was evaluated by modified quantitative PCR (QPCR) method as described previously [66, 67]. Briefly, total DNA was extracted and used as PCR sample. The primer sequences used for mtDNA long segment (16 kb) were mtLongF (5′-GGA CAA ATA TCA TTC TGA GGA GCT-3′) and mtLongR (5′-GGA TTA GTC AGC CGT AGT TTA CGT-3′). The primer sequences for mtDNA short (80 bp) segment were mtShortF (5′-CCAAGGAATTCCCCTACACA-3′) and mtShortR (5′-GAAATTGCGAGAATGGTGGT-3′). The mtDNA long segment and the short segment were amplified using AccuPrime™ Taq DNA Polymerase High Fidelity kit (Life Tech Corp) and separated by agarose gel electrophoresis, respectively. MtDNA long PCR condition was as follows: 94 °C for 11 s, followed by 25 cycles of denaturation at 94 °C for 15 s, annealing and extension at 67 °C for 12 min, final extension at 72 °C for 10 min. MtDNA Short PCR condition was as follows: 94 °C for 6 s, followed by 18 cycles of denaturation at 94 °C for 20 s, annealing and extension at 65 °C for 1 min, and final extension at 72 °C for 10 min. The gels were stained by ethidium bromide and visualized with Alpha Imager, and densitometry analysis performed using Image J software. Lesion frequency per 16 kb of mtDNA was calculated by following equation.

$$ \mathrm{Lesion}\ \mathrm{frequency}\ \mathrm{per}\ 16\ \mathrm{kb}\ \mathrm{of}\ \mathrm{each}\ \mathrm{sample}=\kern0.5em -\mathrm{Ln}\left[\frac{\mathrm{mito}\ \mathrm{long}/\mathrm{mito}\ \mathrm{short}}{\mathrm{average}\ \mathrm{of}\ \left(\mathrm{mito}\ \mathrm{long}/\mathrm{mito}\ \mathrm{short}\ \mathrm{from}\ \mathrm{control}\ \mathrm{group}\right)}\right] $$

### Quantitative real-time RT-PCR

RNA was isolated from cells using TRIzol (Invitrogen 15,596–026) according to the manufacturer’s protocol. 2 μg of RNA was used to convert to cDNA using iScript™ cDNA Synthesis Kit (Bio-Rad 170–8891) according to the manufacturer’s protocol. Quantitative real-time PCR was performed with SYBR Green Mastermix (Life Tech Corp 4,364,346) with the following conditions: 50 °C 2 min, 95 °C 10 min, 95 °C 15 s - > 60 °C 1 min 40 cycles. Results were normalized against an internal control (β-actin).

**Table:** 

becn1 F	ttcaagatcctggaccgagt
becn1 R	cttcctcctggctctctcct
hsc70 F	tgttgctttcaccgacacag
hsc70 R	cgaacctacgtccgatcaga
lamp1 F	aggatcaaccttccccaact
lamp1 R	atgctctggtcacagtcgtg
lamp2 F	ccaaaacatttcctggtgct
lamp2 R	caggtgaatgccccaatagt
lc3b F	cagatcgtctgacccaggac
lc3b R	ccggacatcttccactcttt
oga F	aggttcctgtgcggtgtag
oga R	ttcccatttctgaagccttct
ogt F	agtagtggcggcagtagaag
ogt R	attcccgatgtgccaactc
p62 F	ctgagaaggactcgctcgac
p62 R	tccaaataattctcctcgtca
actin F	gtcgtaccactggcattgtg
actin R	accctcatagatgggcacag

### Proteasome activity assays

We analyzed the proteasome activities in 25–50 μg protein extracts. The assay buffer consists of 50 mM Tris (pH 7.5), 2.5 mM EGTA, 20% glycerol, 1 mM DTT, 0.05% NP-40, and 25 μM substrate. Substrates Ac-nLPnLD-AMC, Bz-VGR-AMC and Suc-LLVY-AMC were from Enzo life science [43, 68]. MG132 was used at a final concentration of 10 μM to block proteasome activities as negative controls. Fluorescence was read at 5 min intervals for 2 h, at an excitation wavelength of 380 nm and an emission wavelength of 460 nM, *n* ≥ 3 per group. Data were activities normalized to total protein and control in the assay.

### Statistical analysis

Data are reported as mean ± SEM. Comparisons between groups were performed with unadjusted, unpaired Student’s t-tests or Wilcoxon for non-parametric comparison or ANOVA and Bonferroni’s Multiple Comparison post-hoc test, as noted. A *p* value of less than 0.05 was considered statistically significant.

## Additional files

Additional file 1: Figure S1. Inhibition of OGA by thiamet G (TG) did not significantly change mitochondrial mass or function. (JPEG 500 kb)

Additional file 2: Figure S2. Proteasome activities are unchanged by OGA inhibition with thiamet G (TG). (JPEG 450 kb)

Additional file 3: Figure S3. Original western blot analyses. Quantification performed by normalizing to the 6 control brain in each gel. (JPEG 202 kb)

Additional file 4: Figure S4. Impact of OGA inhibition by thiamet G (TG) on mRNA and proteins of autophagy genes. (JPEG 587 kb)

Additional file 5: Figure S5. Original western blot analyses of Beclin and p62 WB for the 24 h and 7 day exposure to thiamet G. (JPEG 213 kb)
