# Supplementary figures and images for: O-GlcNAc regulation of autophagy and α-synuclein homeostasis; implications for Parkinson’s disease
Source: Mol Brain. 2017 Jul 19;10:32. doi: 10.1186/s13041-017-0311-1 (PMC5517830; doi:10.1186/s13041-017-0311-1)

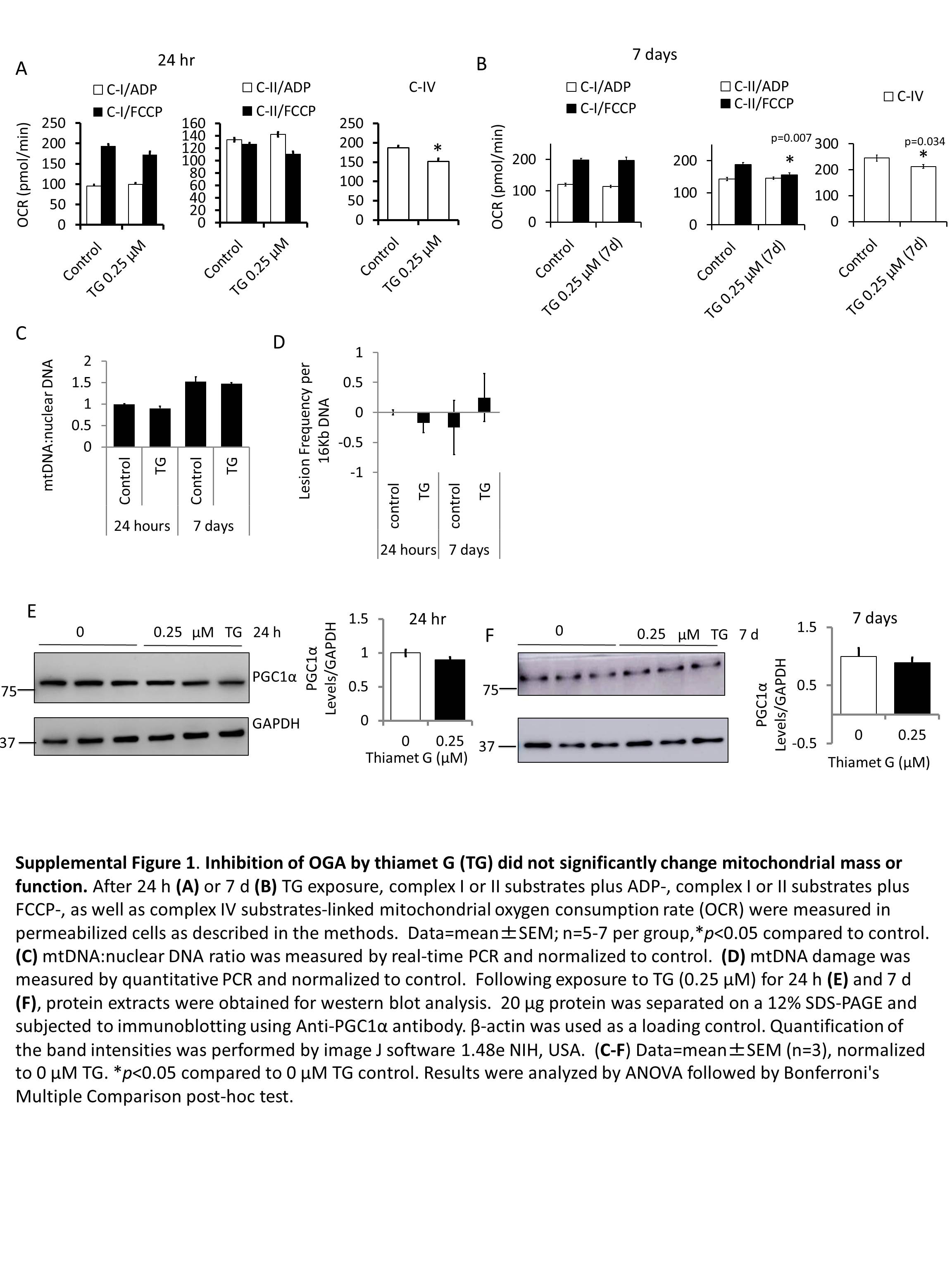

Supplement: Supplementary file 1 — Inhibition of OGA by thiamet G (TG) did not significantly change mitochondrial mass or function. (JPEG 500 kb) [file 13041_2017_311_MOESM1_ESM.jpg]

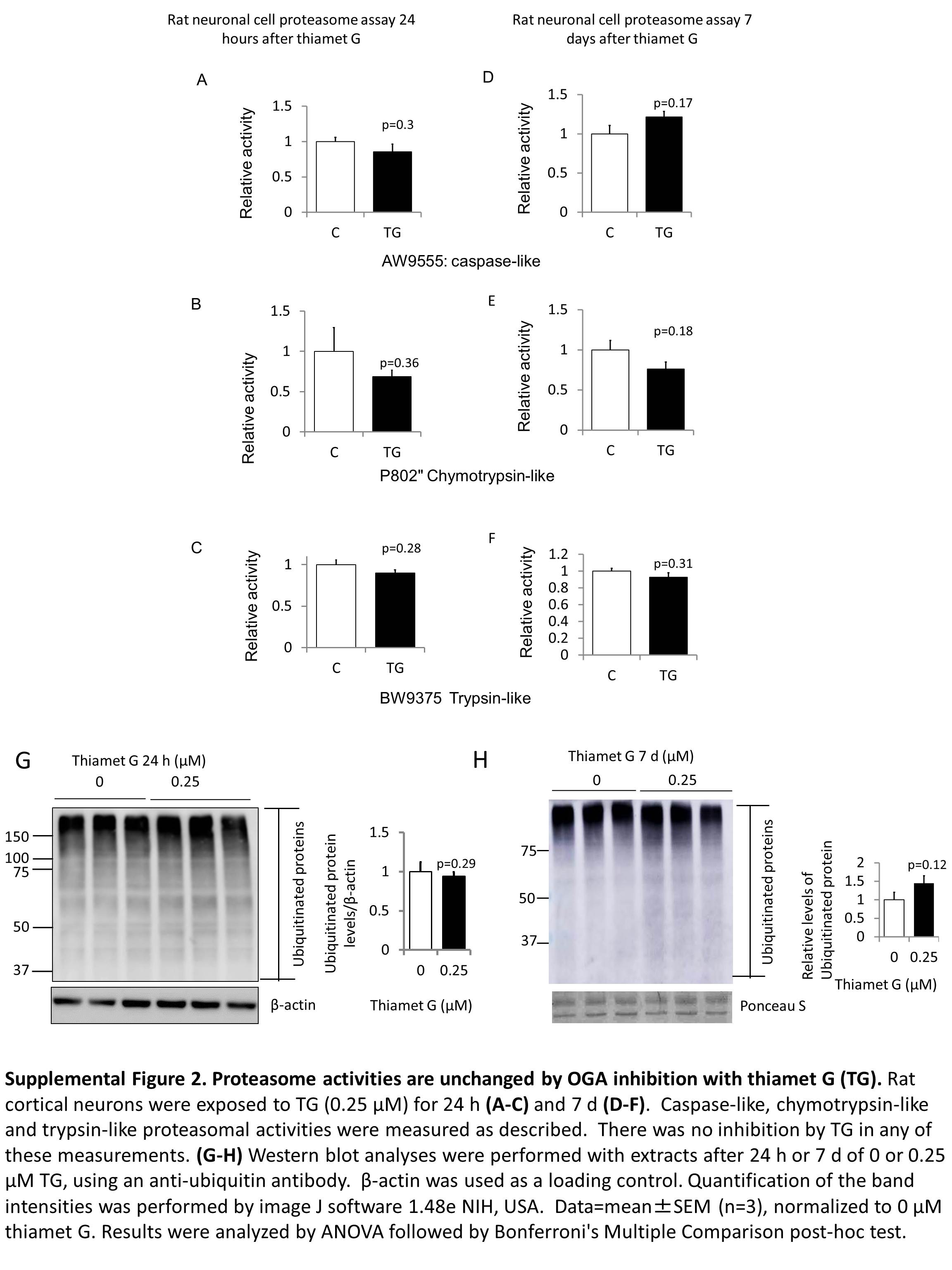

Supplement: Supplementary file 2 — Proteasome activities are unchanged by OGA inhibition with thiamet G (TG). (JPEG 450 kb) [file 13041_2017_311_MOESM2_ESM.jpg]

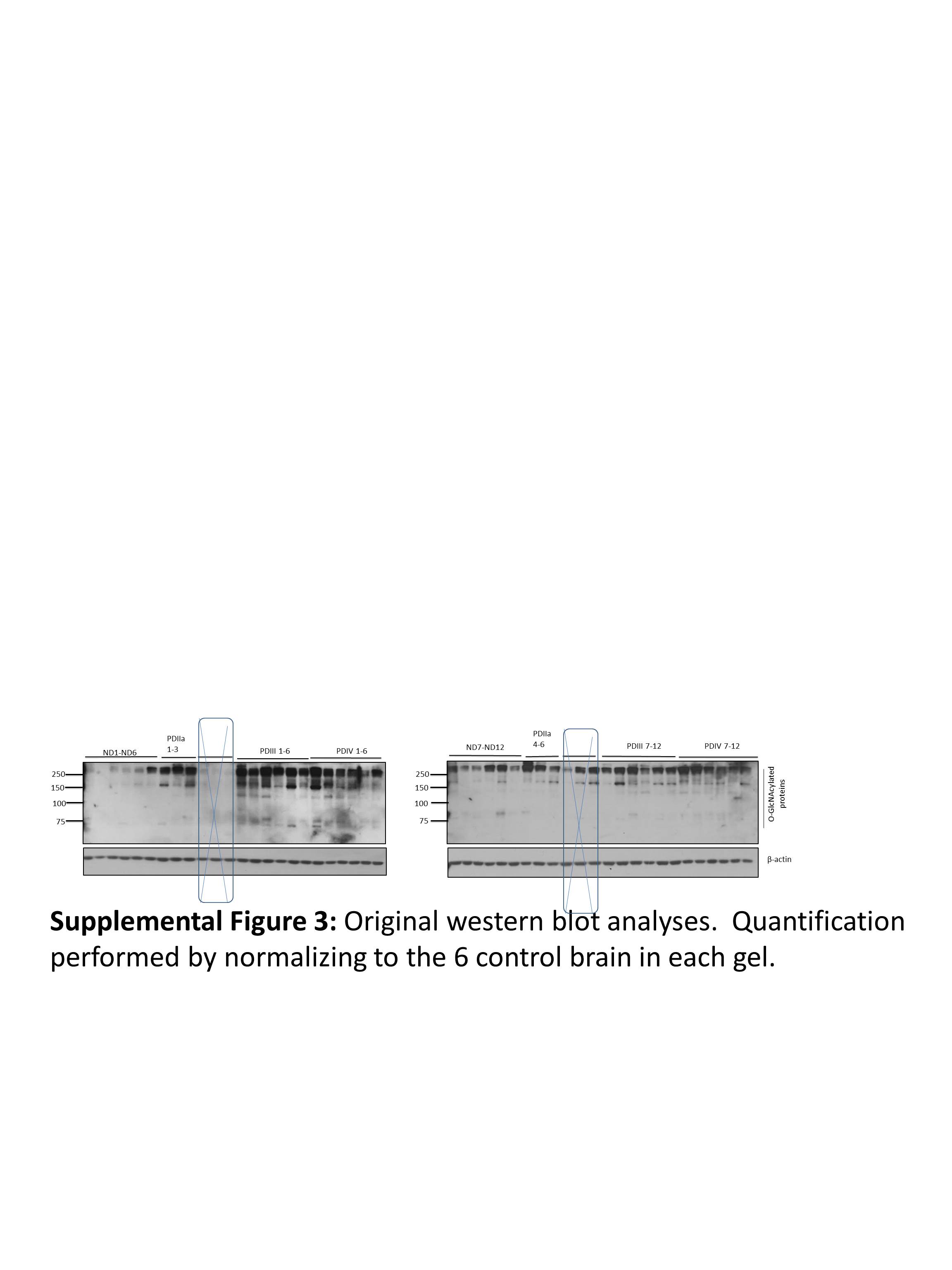

Supplement: Supplementary file 3 — Original western blot analyses. Quantification performed by normalizing to the 6 control brain in each gel. (JPEG 202 kb) [file 13041_2017_311_MOESM3_ESM.jpg]

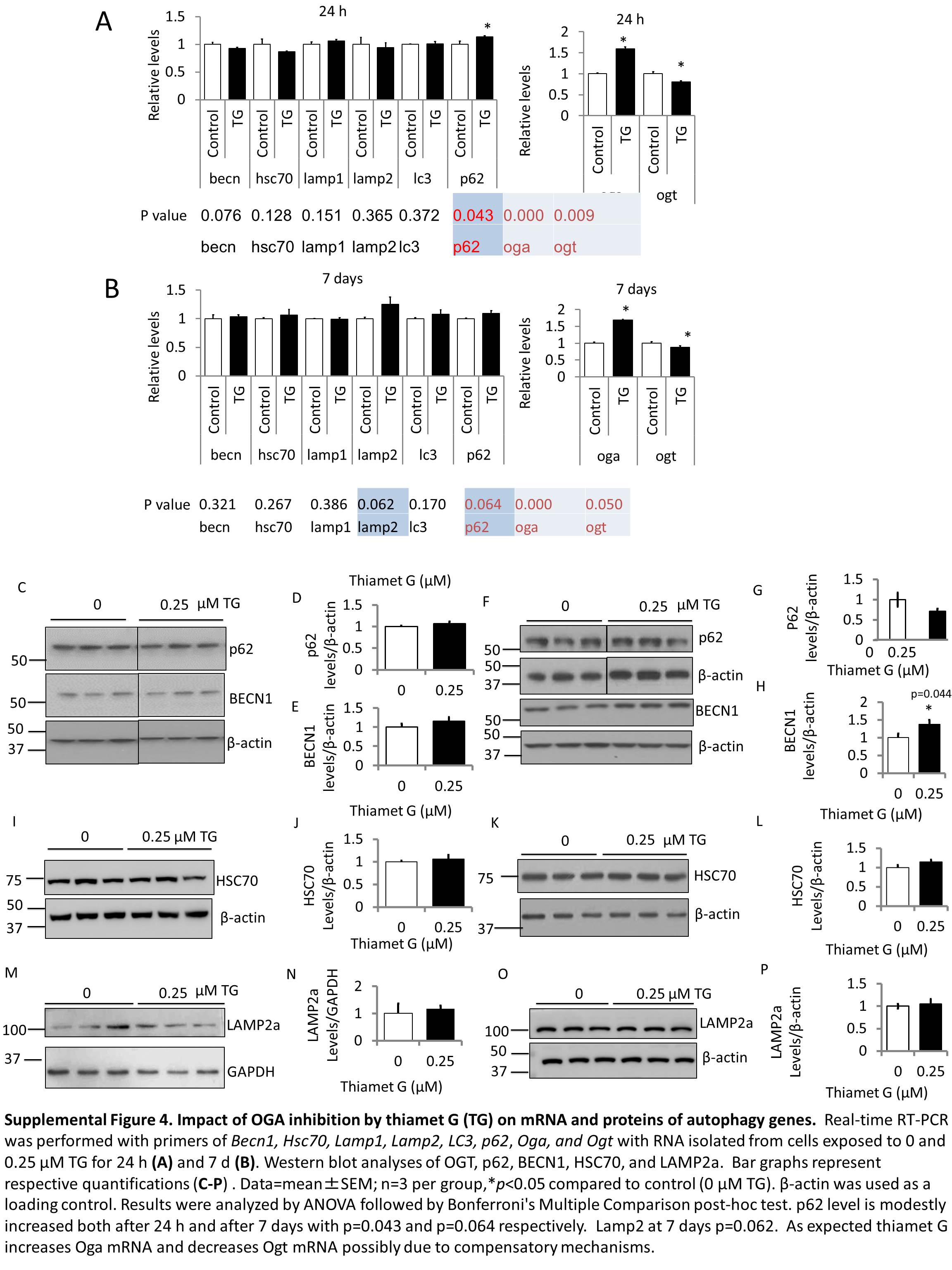

Supplement: Supplementary file 4 — Impact of OGA inhibition by thiamet G (TG) on mRNA and proteins of autophagy genes. (JPEG 587 kb) [file 13041_2017_311_MOESM4_ESM.jpg]

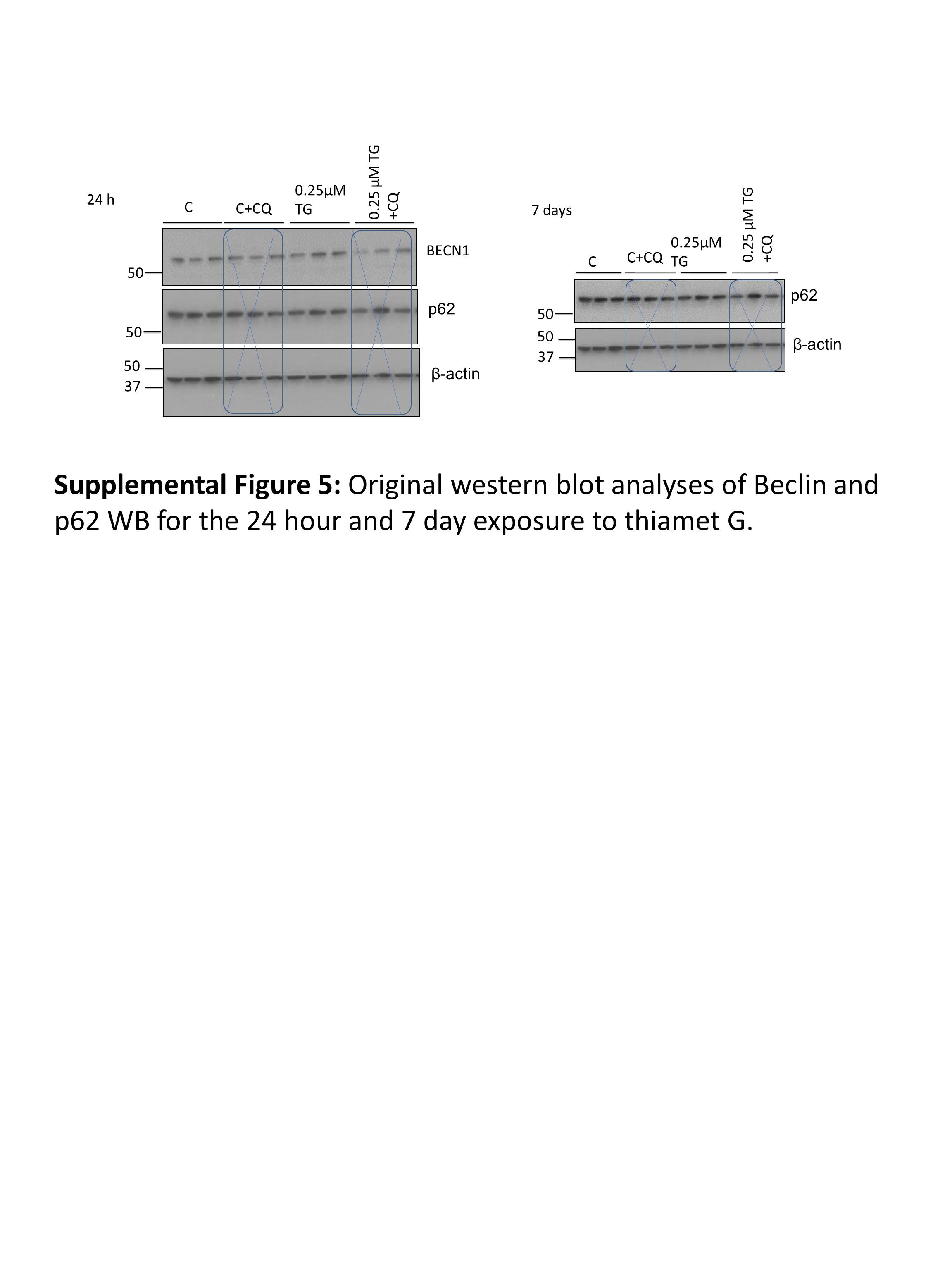

Supplement: Supplementary file 5 — Original western blot analyses of Beclin and p62 WB for the 24 h and 7 day exposure to thiamet G. (JPEG 213 kb) [file 13041_2017_311_MOESM5_ESM.jpg]
